# Improving the solubility of single domain antibodies using VH‐like hallmark residues

**DOI:** 10.1002/pro.70189

**Published:** 2025-06-16

**Authors:** Yuta Uto, Makoto Nakakido, Takanori Yokoo, Jorge Fernandez‐Perez, Kevin Entzminger, Toshiaki Maruyama, C. J. Okumura, Daisuke Kuroda, Jose M. M. Caaveiro, Kouhei Tsumoto

**Affiliations:** ^1^ Department of Chemistry and Biotechnology, School of Engineering The University of Tokyo Tokyo Japan; ^2^ Department of Bioengineering, School of Engineering The University of Tokyo Tokyo Japan; ^3^ Abwiz Bio Inc. San Diego California USA; ^4^ Research Center for Drug and Vaccine Development National Institute of Infectious Diseases Tokyo Japan; ^5^ Department of Protein Drug Discovery, Graduate School of Pharmaceutical Sciences Kyushu University Fukuoka Japan; ^6^ Medical Device Development and Regulation Research Center, School of Engineering The University of Tokyo Tokyo Japan; ^7^ The Institute of Medical Science The University of Tokyo Tokyo Japan

**Keywords:** additive, nanobody, physicochemical analysis, protein solubility, single domain antibody, solubility prediction, VH, VHH

## Abstract

Single domain antibodies (sdAbs) can be generated from variable regions of heavy‐chain antibodies, which lack light chain and CH1 region. They have attracted attention due to their small size and molecular characteristics. Hydrophilic hallmark amino acids at framework region 2 (FR2) are key residues involved in the solubility of sdAbs. Nevertheless, previous studies reported that several sdAbs with human VH‐like hydrophobic hallmark residues were soluble in a monomeric state and suggested that solubility also depends on the amino acid sequences in the complementarity‐determining region. In this study, we obtained two sdAbs (sdAb A and B) with VH‐like hallmark residues and low solubility from an alpaca immune library. We introduced VHH‐like mutations (V37Y, G44E, L45R, W47L) into the hallmark residues in FR2 of both sdAb A and B. We were able to prepare sdAb A as a monomer without an additive in the buffer, but sdAb B was polydispersed when arginine was not added to the buffer. We also predicted the hydrophobicity of the sdAb B surface by spatial aggregation propensity calculations and identified W99 as the residue responsible for its low solubility. Subsequently, we obtained the sdAb B mutant as a monomer by introducing the W99A mutation. We characterized the engineered sdAbs using structural, physicochemical, and biophysical analyses and found that the solubility‐improved sdAbs retained their functionality. Our findings can be applied to improving the solubility of sdAbs even in the absence of structural information.

## 1 INTRODUCTION

Antibodies (Abs) recognize antigens with high specificity and affinity. To date, Abs have been widely used to treat various diseases and as research tools. Among the various subtypes of Abs, heavy‐chain antibodies (HCAbs) are camelid‐specific Abs that were first described in 1993 (Hamers‐Casterman et al., 1993). HCAbs have the unique characteristic of lacking light chains.

Single domain antibodies (sdAbs), which can be generated by extracting variable regions of HCAbs (Hamers‐Casterman et al., 1993; Kunz et al., 2023), offer numerous advantages over conventional Abs due to their small size (~15 kDa) and single‐domain structure. For example, sdAbs can be expressed by *Escherichia coli* (Arbabi‐Ghahroudi et al., 2005), thus production costs are lower compared to those of conventional immunoglobulin G (IgG) (Jovčevska & Muyldermans, 2020; Kunz et al., 2023). Also, previous studies have shown that several sdAbs can be connected easily via amino acid linkers, with each retaining its binding activity (Jindal et al., 2024; Weinstein et al., 2022). Furthermore, sdAbs have high tissue permeability (Bannas et al., 2015; Debie et al., 2020; Muruganandam et al., 2002). Several studies have suggested that sdAbs tend to adopt unique antigen binding modes compared to conventional Abs because they contain fewer complementarity‐determining region (CDR) loops (de Genst et al., 2006; Muyldermans et al., 2001). Due to these molecular properties, sdAbs have attracted a great deal of attention.

One of the most distinctive features of sdAbs is the presence of hydrophilic amino acids at framework region 2 (FR2) (positions 37, 44, 45, and 47; Chothia numbering (Chothia & Lesk, 1987)). Amino acids corresponding to these positions in conventional Abs are hydrophobic and allow interaction with the VL domain (Kunz et al., 2023). Several reports have suggested that sdAbs acquire high colloidal stability due to their hydrophilic residues (Conrath et al., 2005; Riechmann, 1996). However, Ward et al. (1989) suggested that sdAbs with VH‐like hallmark 4 residues (V37, G44, L45, W47; Chothia numbering (Chothia & Lesk, 1987)) can lead to aggregation due to exposed hydrophobic surfaces in solution (Ward et al., 1989). Indeed, resurfacing the framework residues of sdAbs with VH‐like hallmark residues has been investigated, and the tested mutations significantly affected sdAb solubility (Riechmann, 1996; Vincke et al., 2009). Other studies (Jespers et al., 2004; Soler et al., 2021) showed that even sdAbs with human VH‐like hallmark residues in FR2 could be stable in solution in a monomeric state, but the solubility was highly dependent on the amino acid sequences in the CDR.

Various methods have been utilized to improve the solubility of proteins (Ebo et al., 2020; Johnson et al., 2014). Additives in the buffer can improve solubility without changing protein sequences (Leibly et al., 2012). For example, arginine (Arg) is a well‐known aggregation suppressor (Johnson et al., 2014) that functions by reducing protein–protein interactions (Tsumoto et al., 2005). An alternative way to increase the solubility of proteins with low solubility is to introduce mutations of specific amino acids (Trevino et al., 2008).

To effectively improve the colloidal stability of proteins, structure‐based computational designs have been developed (Chennamsetty et al., 2009; Sormanni et al., 2015; Zalewski et al., 2024). Spatial aggregation propensity (SAP) is a simulation that predicts the hydrophobic regions of therapeutic proteins, such as IgG (Chennamsetty et al., 2009). The SAP calculation assesses the exposure of hydrophobic residues by averaging data from snapshots obtained in molecular dynamics (MD) simulations conducted with explicit water (Chennamsetty et al., 2009; Kuroda et al., 2012). Most previous applications have focused on improving the solubility of Abs that already possess a certain level of colloidal stability using available crystal structures (Chennamsetty et al., 2009; Sormanni et al., 2015). However, a subsequent work suggested that SAP calculation could also be applied to predicted structures (Chennamsetty et al., 2010).

In this study, we attempted to establish a strategy to improve the solubility of sdAbs with human VH‐like hallmark 4 residues. Using model sdAbs isolated from an alpaca immune library, we analyzed the effect of additives during the purification steps and also designed mutants based on the hydrophobicity prediction obtained by SAP calculation using predicted model structures. We then experimentally prepared the designed mutants and evaluated their physicochemical properties. Based on the results, we suggest a strategy to improve the solubility of sdAbs even in the absence of structural data.

## 2 RESULTS

### 2.1 Acquisition of anti‐neuroligin (Nlgn)2 sdAbs

**FIGURE 1:**
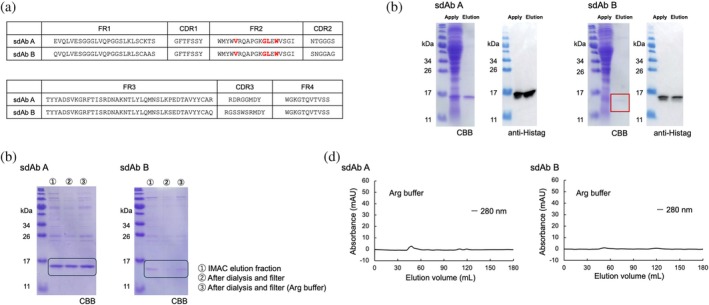
Expression and purification of sdAb A and B. (a) The amino acid sequences of sdAb A and B. Framework region (FR) and complementarity determining region (CDR) were defined using the Chothia numbering method. (b) The results of SDS‐PAGE and western blotting analyses after immobilized metal affinity chromatography (IMAC). SDS‐PAGE gel was stained with Coomassie brilliant blue (CBB). (c) The results of SDS‐PAGE to compare the amount of soluble protein after dialysis and filtration. (d) Chromatograms of size exclusion chromatography (SEC).

Neuroligin (Nlgn) family proteins are post‐synaptic cell adhesion molecules. Nlgn2 plays a key role as a central organizer of inhibitory synapses (Ali et al., 2020). To date, no sdAbs targeting Nlgn2 have been reported. To obtain sdAbs against Nlgn2, we prepared the extracellular domain of Nlgn2 as a recombinant protein and immunized an alpaca with it. After confirmation of the antibody titer in serum, we extracted RNA from lymphocytes and constructed a phage display‐based immune library. Subsequently, we isolated sdAb A and B by bio‐panning via phage display from the immune library (Figure 1a).

The amino acid sequences in FR2 at the VHH hallmark positions of our sdAbs (V37, G44, L45, W47) were the same as those of human VH. Although a previous study showed that some sdAbs from immune libraries have the same hallmark residues as human VH and can be purified in a monomeric state, the solubility was highly dependent on the CDR sequences (Soler et al., 2021). Therefore, we assessed the solubility of our sdAbs.

We expressed the sdAbs as recombinant proteins using an *E. coli* expression system. Expressed sdAbs were purified by immobilized metal affinity chromatography (IMAC) (Figure 1b), and both sdAbs were eluted as soluble protein. Before the final purification by size exclusion chromatography (SEC), we dialyzed eluted proteins against SEC buffer followed by filtration through a 0.2 μm pore‐sized filter. We confirmed the existence of visible aggregations that were trapped in this filter. Because soluble proteins still remained after filtering, especially in the presence of Arg (Figure 1c), we subjected the sample to SEC using Arg buffer. However, sdAbs were not eluted by SEC (Figure 1d), suggesting that proteins were absorbed on the column resin.

### 2.2 Introduction of FR2 mutations

**FIGURE 2:**
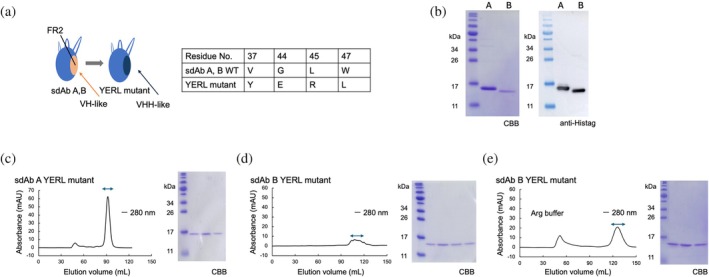
Design of the VHH‐mimic framework region (FR). (a) Details about the mutations in FR2. Chothia numbering was used. (b) Results of SDS‐PAGE and western blotting analysis after immobilized metal affinity chromatography (IMAC). (c) Chromatogram of the sdAb A FR2 YERL mutant obtained from size exclusion chromatography (SEC) and the results of SDS‐PAGE analysis after SEC. (d) SEC chromatogram and SDS‐PAGE results for the sdAb B FR2 YERL mutant. (e) Purification of the sdAb B FR2 YERL mutant in the presence of 200 mM arginine (Arg).

To address the aggregation of sdAbs, we introduced mutations into FR2 residues. VHH, which often has a high solubility, conserves F/Y37, E/Q44, R45, and G/L/F47 in FR2. Also, a previous study showed the clear distinction of these sequences depending on the length of CDR3 loops (Kuroda & Tsumoto, 2023). In the case of VHH possessing long CDR3 (bent conformation), the bulkier residues (F37 and F47) are preferred and intramolecular interactions between CDR3 and FR2 are often observed (Kinoshita et al., 2022; Kuroda & Tsumoto, 2023). On the contrary, VHH hallmark residues (Y37, E44, R45, L47) in FR2 are thought to be compatible with VHHs bearing short and extended CDR3 loops in which CDR3 and FR2 do not interact with each other (Kinoshita et al., 2022; Kuroda & Tsumoto, 2023), and sdAb A and B have short CDR3 loops (8 and 10 residues in the Chothia definition (95–102), respectively, Figure 1a). We employed site‐directed mutagenesis and introduced all four VHH‐like mutations (V37Y, G44E, L45R, W47L) into the hallmark residues in FR2 of both sdAb A and B (denoted as sdAb FR2 YERL mutants) (Figure 2a) and prepared the FR2 YERL mutants as recombinant proteins. We expressed each sdAb FR2 YERL mutant in *E. coli*, purified the supernatant by IMAC, and confirmed the presence of soluble proteins in the elution fraction for each mutant (Figure 2b).

The sdAb A FR2 YERL mutant was eluted as a monodispersed peak in subsequent SEC (Figure 2c), whereas the sdAb B FR2 YERL mutant showed a broad polydispersed peak (Figure 2d). Although a monodispersed elution of the sdAb B FR2 YERL mutant appeared after the addition of Arg into SEC buffer, this mutant was eluted at more than one column volume (Figure 2e), suggesting that this mutant interacted with the column resin. In summary, although the mutations of FR2 YERL improved the solubility of both sdAbs, sdAb B retained the hydrophobic surface and therefore it was difficult to purify it as a monomer without the addition of the additive.

### 2.3 Prediction and remodeling of the hydrophobic surface

**FIGURE 3:**
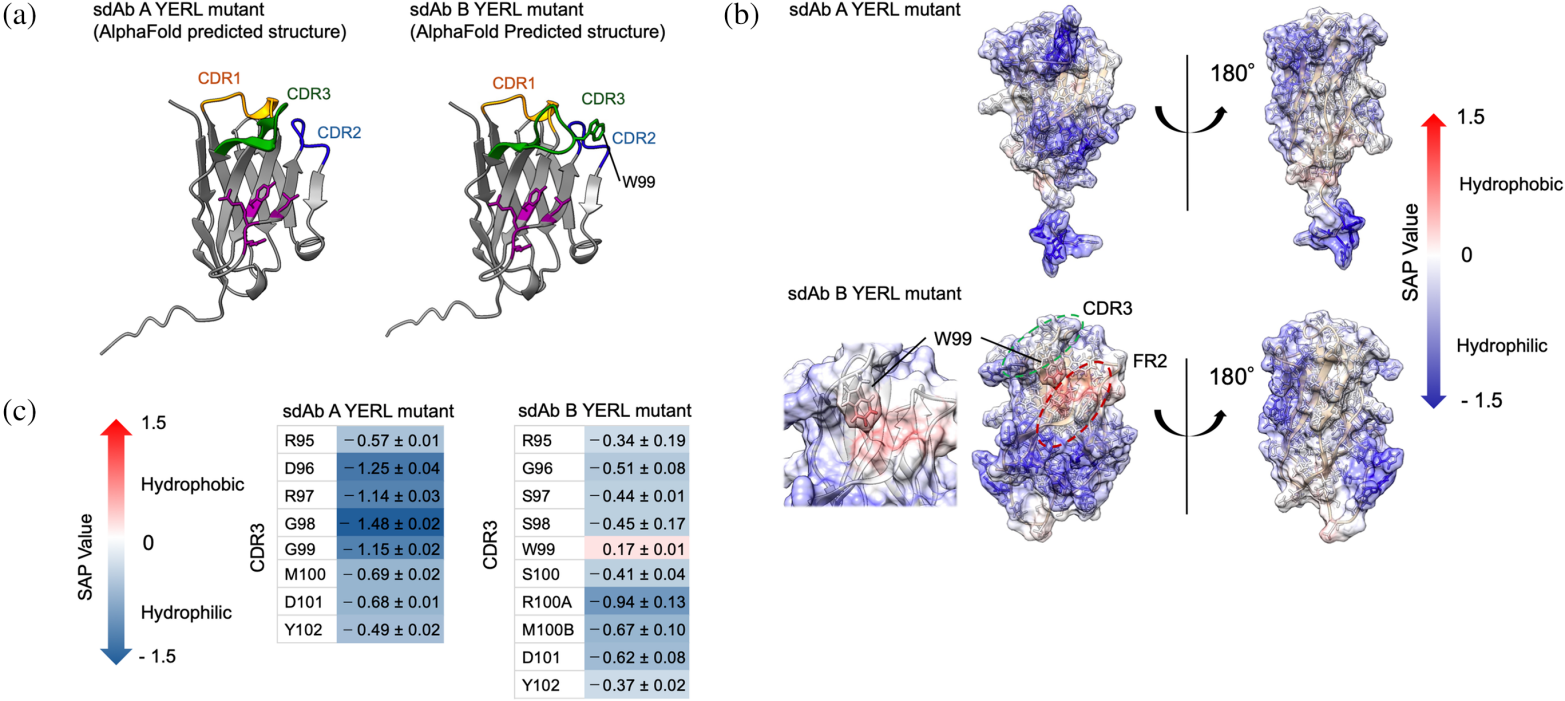
Prediction of colloidal stability by spatial aggregation propensity (SAP) calculation. (a) Predicted structure of sdAb FR2 YERL mutants. The mutated residues and CDRs are highlighted in purple, orange, blue, and green, respectively. (b) SAP mapped structures of the sdAb FR2 YERL mutants. W99 of sdAb B is located in the middle of CDR3. The structures (left) are in a similar orientation to that in panel (a). (c) SAP values per residues in CDR3. The average and standard error values from three independent simulations are shown.

Because sdAb A and sdAb B share the same FR2 YERL sequence but differ in the sequences and lengths of their CDR3 regions, the difficulty in purifying the sdAb B FR2 YERL mutant as a monomer likely is due to the differences in their CDR3 sequences and, consequently, their structures. To evaluate surface hydrophobicity in the context of three‐dimensional structures, we prepared model structures of the FR2 YERL mutants using AlphaFold2 (Mirdita et al., 2022) (Figure 3a). We then conducted MD simulations using the predicted structures of both sdAb A and B, followed by SAP calculations of the centroid structures obtained through clustering the MD trajectories. SAP calculation gives the dynamically exposed hydrophobicity of a certain patch on the protein surface by analyzing spherical zones around each atom in a protein structure, combining solvent accessible area (SAA) measurements (Chennamsetty et al., 2009; Chennamsetty et al., 2010).

Overall, the surface of sdAb A was less hydrophobic than that of sdAb B according to the SAP mapped structures (Figure 3b), which is consistent with the results of the SEC analysis. In particular, the CDR3/FR2 region of sdAb B was predicted to be a hydrophobic region, which is illustrated by the red color in Figure 3. We next focused on the CDR3 and compared the SAP value per residue between sdAb FR2 YERL mutants. Because W99 had the highest SAP value among the residues in CDR3, we hypothesized that W99 of sdAb B is a key residue for surface hydrophobicity. Therefore, we prepared an sdAb B mutant that possesses the W99A mutation (denoted as sdAb B YERLA mutant, the terminal A corresponds to W99A).

**FIGURE 4:**
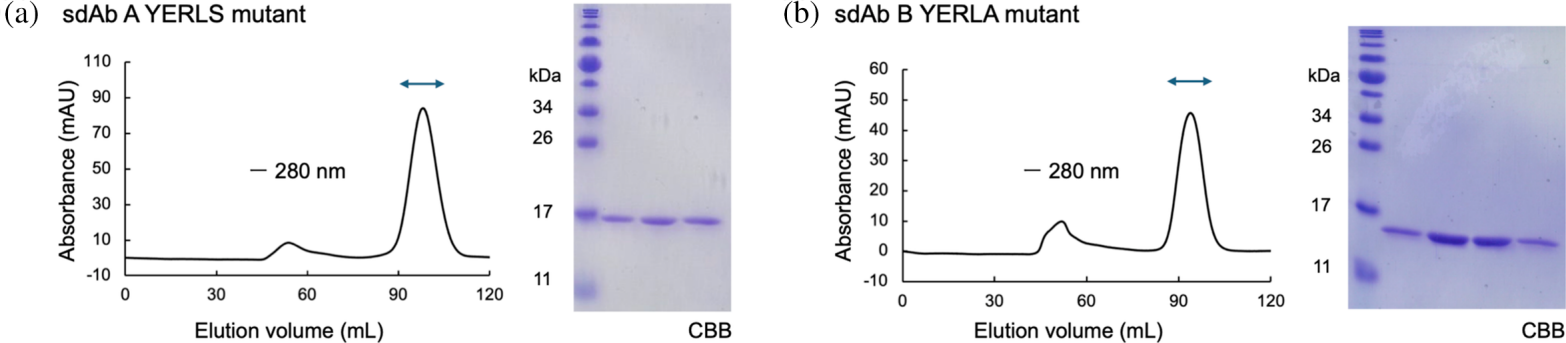
Purification of sdAb A and B mutants based on spatial aggregation propensity (SAP) calculation. The chromatograms and the results of SDS‐PAGE analysis after size exclusion chromatography (SEC) are shown. Purification of the (a) sdAb A YERLS mutant and (b) sdAb B YERLA mutant.

We expressed sdAb B YERLA following the same procedure we used for the other sdAbs, purified it by IMAC, and then conducted SEC. The elution volume in SEC indicated that sdAb B YERLA was eluted as a monomer, even without the addition of Arg. This result suggested that the W99A mutation successfully reduced the surface hydrophobicity of sdAb B and improved colloidal stability (Figure 4b).

To further enhance the solubility of sdAb A, we explored the effects of additional mutations to reduce surface hydrophobicity. sdAb A possesses leucine at position 11, whereas the corresponding residue in camelid VHH is highly conserved as serine. The L11 residue normally interacts with the CH1 domain on the IgG antibody; this domain lacks the camelid heavy chain antibody (Hamers‐Casterman et al., 1993) and the exposed L11 residue should be hydrophobic. Taken together with a previous study suggesting that mutation of leucine to serine would improve the solubility of VHH (Muyldermans et al., 1994), we introduced the L11S mutation in the sdAb A FR2 YERL mutant (denoted as sdAb A YERLS, the terminal S corresponds to L11S) to investigate the effects of this mutation on solubility. We expressed and purified sdAb A YERLS by IMAC, conducted SEC, and confirmed that sdAb A YERLS was eluted in a monomeric state (Figure 4a).

### 2.4 Characterization of sdAb solubilizing mutants

**FIGURE 5:**
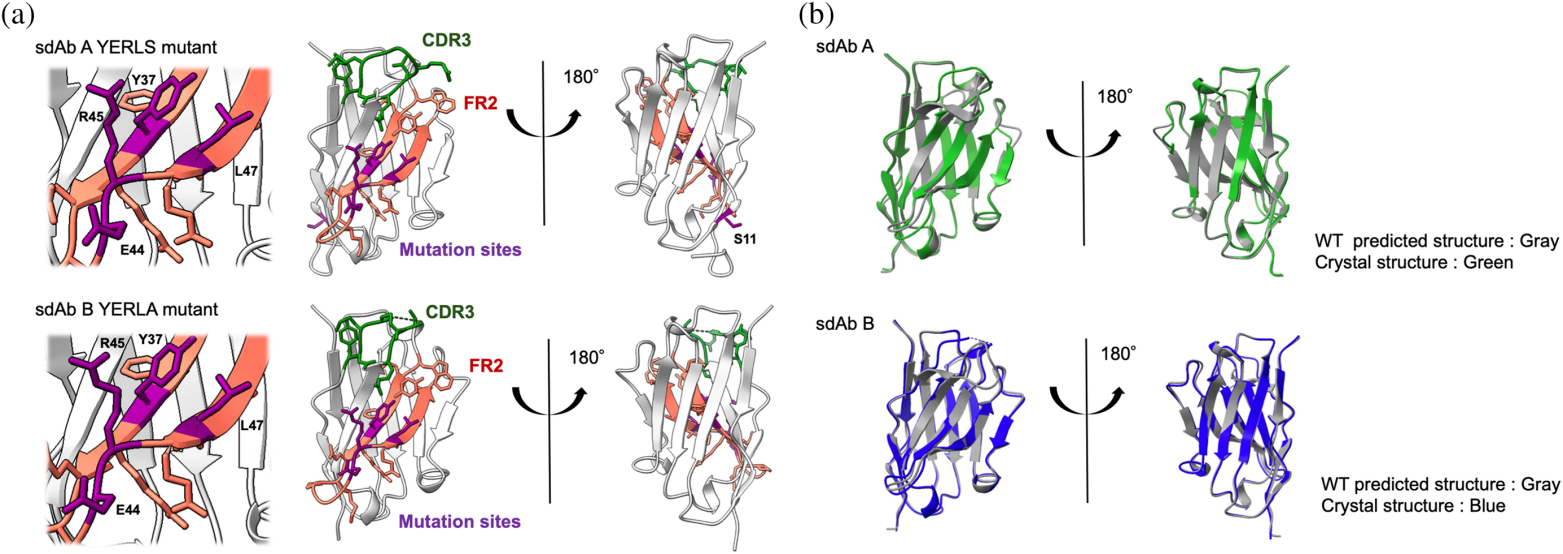
Crystal structures of sdAb A and B mutants. (a) Structure of sdAb A YERLS and sdAb B YERLA mutant. Framework region 2 (FR2) complementarity determining region 3 (CDR3), and mutation sites are highlighted in orange, green, and purple, respectively. (b) Superposition of crystal structures with the predicted structure of wild type sdAbs.

To confirm that the mutations did not alter the overall structure of the sdABs, we conducted X‐ray crystallographic analysis for both sdAb A YERLS and sdAb B YERLA. The crystal structures of these mutants were determined at 1.57 Å and 1.62 Å resolution, respectively (Figure 5a, Table 1). We were unable to assign the residues in CDR3 of the sdAb B YERLA mutant structure due to the weak electron density. This result suggests high flexibility of the CDR3 loop, even though its length is relatively shorter (10‐residues) than the average CDR3 length. Nevertheless, the overall structure for each mutant resembles that of the typical sdAb structure.

**TABLE 1:** Data collection and refinement statistics.

Data collection	sdAb A YERLS mutant	sdAb B YERLA mutant
Space Group	P 2_1_ 2_1_ 2_1_	P 3 2 1
Unit cell		
a, b, c (Å)	52.4, 113.3, 113.9	77.9, 77.9, 45.3
α, β, γ (°)	90.0, 90.0, 90.0	90.0, 90.0, 120.0
Resolution (Å)	47.6–1.57 (1.60–1.57)	45.3–1.62 (1.65–1.62)
Wavelength	1.0000	1.0000
Observations	414,341 (19,899)	203,405 (10,052)
Unique reflections	93,118 (4509)	20,480 (990)
*R*_merge_.	0.048 (0.730)	0.075 (0.898)
*R*_p.i.m_.	0.025 (0.392)	0.025 (0.295)
CC_1/2_	0.999 (0.695)	0.998 (0.797)
*I*/*σ (I)*	17.2 (2.2)	16.3 (2.6)
Multiplicity	4.4 (4.4)	9.9 (10.2)
Completeness (%)	97.9 (96.6)	100.0 (100.0)
Refinement statistics		
Resolution (Å)	47.6–1.57	45.3–1.62
*R*_*work*_ / *R*_*free*_ (%)	13.6 / 17.0	16.0 / 20.0
No. protein units	4	1
No. atoms		
sdAb	3791	905
Other	60	33
Water	584	16
B‐factor (Å^2^)		
sdAb	22.1	33.1
Other	40.7	45.2
Water	34.4	42.5
Ramachandran plot		
Preferred (%)	94.7	92.6
Allowed (%)	5.3	7.4
Outliers (%)	0.0	0.0
RMSD Bond (Å)	0.006	0.006
RMSD Angle (°)	1.51	1.44
PDB entry code	9L1K	9L1J

*Note*: Statistical values given in parenthesis refer to the highest resolution bin.

We then compared the determined structures with the structures of the wild type (WT) sdAb predicted by AlphaFold2 for both sdAb A and B (Figure 5b). When FR structures were superposed, the root‐mean‐squared deviation (C α‐RMSD) values between the crystal structure of mutants and predicted WT structures were 0.58 Å and 0.40 Å for sdAb A and B, respectively. Collectively, these results showed that the mutations in FR2 and CDR3 did not alter the overall architecture of sdAb A and B. The noticeable difference in the CDR3 of sdAb A compared to that of sdAb A WT AlphaFold2‐predicted structure (Cα‐RMSD was 3.67 Å) is most likely due to the limited prediction accuracy of AlphaFold2 and the effect of the W99A mutation. Previous benchmark studies have demonstrated that, on average, RMSD values exceed 2 Å and can be higher than 5 Å when comparing AlphaFold2‐predicted structures to crystal structures (Chen et al., 2024; Ruffolo et al., 2023). Despite the current limitations in accurately predicting CDR3 structures of sdAbs, our results suggest that the strategy proposed herein is applicable even when using predicted antibody structures.

**FIGURE 6:**
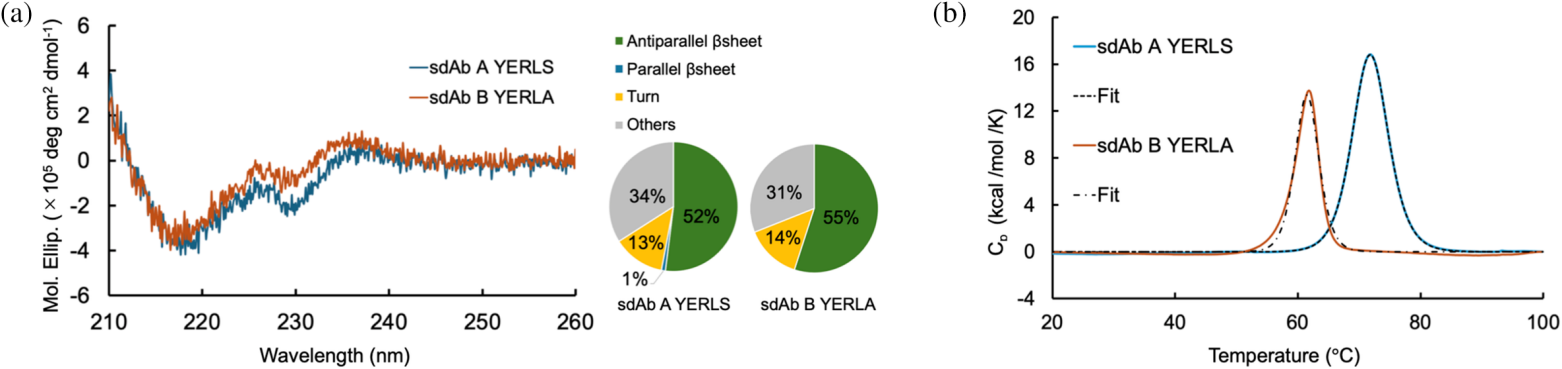
Physicochemical analyses of sdAb mutants. (a) Circular dichroism (CD) spectra and secondary structural components of sdAb mutants. The spectra for sdAb A YERLS and sdAb B YERLA are shown in blue and orange, respectively. (b) Thermograms of differential scanning calorimetry (DSC) analyses for sdAb mutants. The thermogram for sdAb A YERLS and sdAb B YERLA are shown in blue and orange, respectively.

We also performed physicochemical analyses to assess secondary structures in solution. The circular dichroism (CD) spectra resemble those of previously reported sdAbs (Kinoshita et al., 2022). We also analyzed secondary structural components by using the BeStSel webserver (Kardos et al., 2025; Micsonai et al., 2022) (Figure 6a). These results suggested that sdAb solubilizing mutants maintained their secondary structures, which is consistent with the crystal structure results (Figure 6a). We also evaluated the thermal stabilities of the sdAbs. Differential scanning calorimetry (DSC) analysis of the sdAbs revealed that the melting temperature (*T*_m_) of sdAb A YERLS was higher than that of sdAb B YERLA (Figure 6b, Table 2). Considering a previous study suggesting that CDRs contribute greatly to the thermal stability of sdAbs (Micsonai et al., 2022), our DSC results also suggest the decrease in thermal stability of sdAb B YERLA would be due to the flexibility of CDR3. Nevertheless, the observed *T*_m_ values were comparable to the results of previous conventional sdAb thermal stability studies (Kardos et al., 2025).

**TABLE 2:** Melting temperature (*T*_m_) and ΔH values of sdAb solubilizing mutants.

	*T*_m_ (°C)	ΔH (kcal/mol)
sdAb A YERLS	71.8 ± 0.1	130 ± 1.2
sdAb B YERLA	61.5 ± 0.0	70.6 ± 1.7

*Note*: Averages and standard deviations of three independent measurements are shown.

**FIGURE 7:**
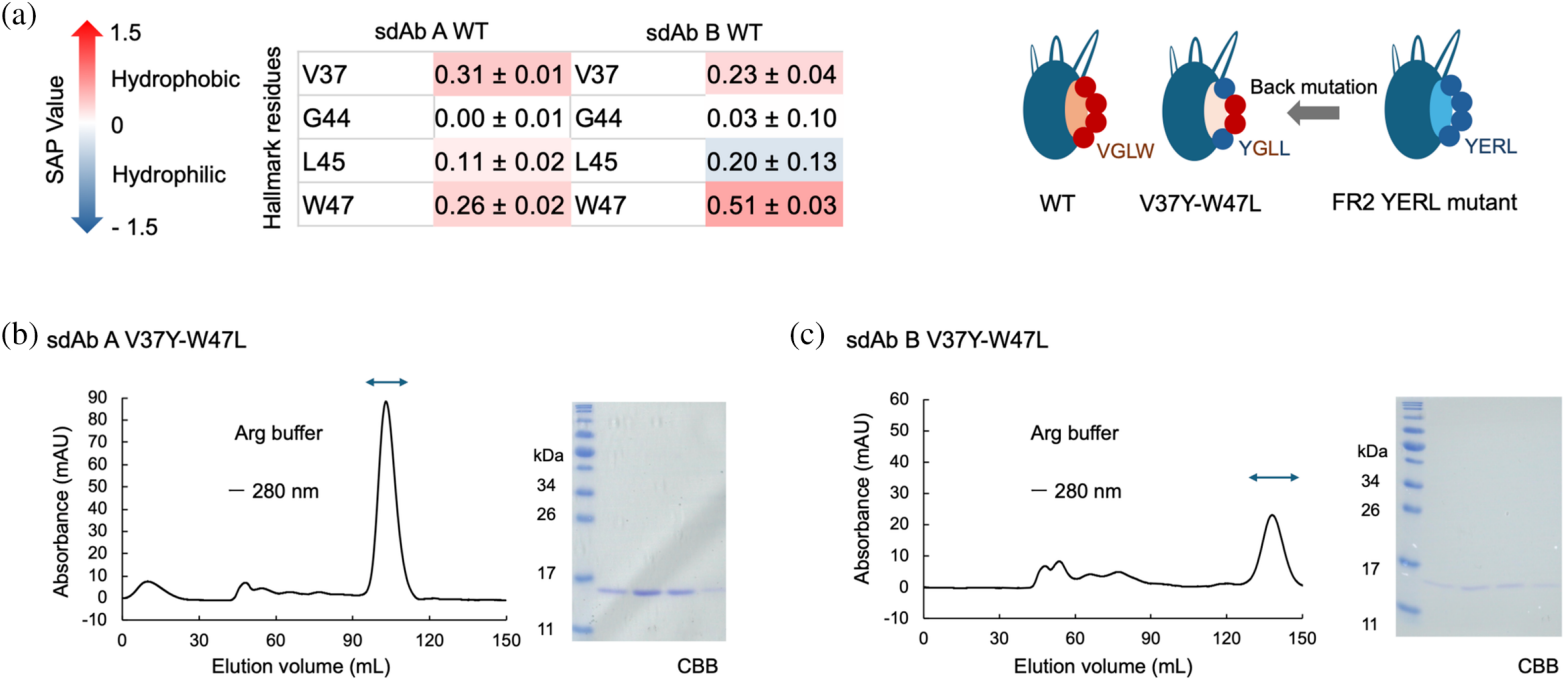
Design of back mutation and purification of sdAb A and B V37Y‐W47L in the presence of 200 mM arginine (Arg). (a) SAP values per residue for hallmark residues. The average and standard error values from three independent simulations are shown. (b) Chromatogram of the sdAb A V37Y‐W47L obtained from size exclusion chromatography (SEC) and the results of SDS‐PAGE analysis after SEC. (c) SEC chromatogram and SDS‐PAGE results for the sdAb B V37Y‐W47L.

To assess the impact of the mutations on thermal stability, we prepared additional mutants in which the hall mark residues in FR2 were partially back‐mutated and performed DSC analysis. To determine the residues to be back‐mutated, we calculated SAP values for the hall mark residues of sdAb WT predicted models in the same method as that of FR2 YERL mutant (Figure 7a, Figure S2) and we selected G44 and L45, which appear to have less contribution to the hydrophobicity. We introduced back mutations and prepared sdAb A and B V37Y‐W47L mutants, which possess two mutations in FR2, following the same procedure as the other sdAbs. The sdAb V37Y–W47L mutants showed monodispersed elution in SEC with Arg‐containing buffer (Figure 7b,c) although sdAb B V37Y–W47L mutant was eluted at more than one column volume, as with sdAb B FR2 YERL mutant.

**FIGURE 8:**
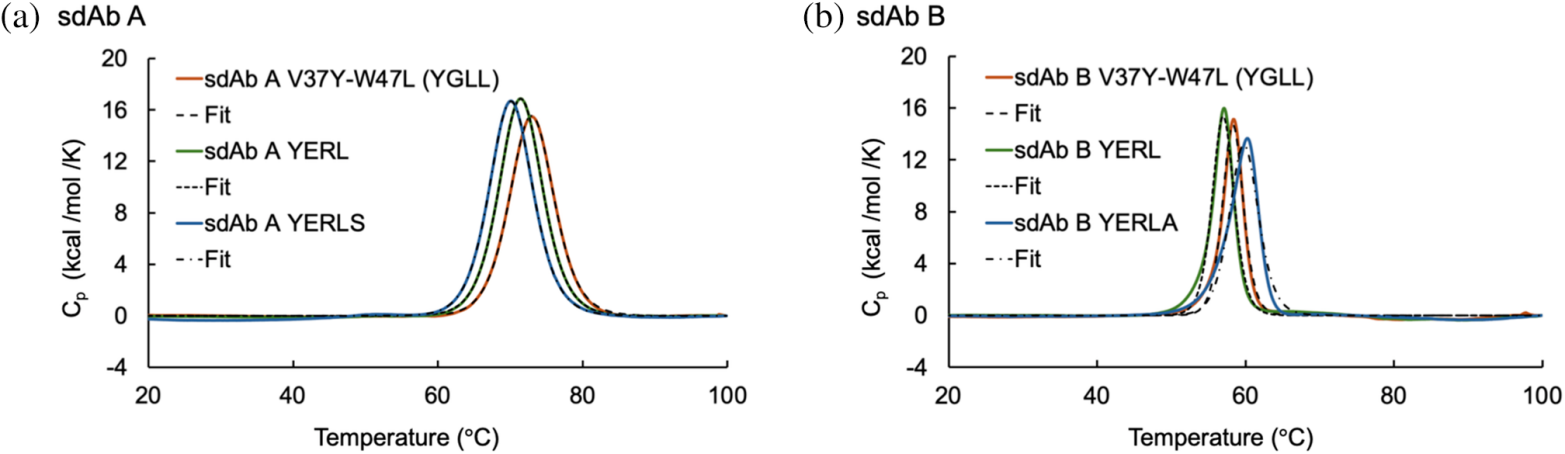
Physicochemical analysis of sdAb mutants. Thermograms of differential scanning calorimetry (DSC) analyses for sdAb mutants in Arg containing buffer. (a) The thermogram for sdAb A V37Y‐W47L, FR2 YERL mutant, and sdAb A YERLS are shown in orange, green, and blue, respectively. (b) The thermogram for sdAb B V37Y‐W47L, FR2 YERL mutant, and sdAb B YERLA are shown in orange, green, and blue, respectively.

We conducted DSC analysis for the partially back‐mutated mutants together with sdAb A FR2 YERL mutant and sdAb B FR2 YERL mutant in the presence of Arg and compared with the *T*_m_ values of fully mutated sdAb A YERLS and sdAb B YERLA, respectively. The results revealed that the mutations in FR2 slightly decreased thermal stability for both sdAb A and B (Figure 8a,b). Although mutations in FR resulted in small destabilization, the observed *T*_m_ values were comparable with those of previously characterized conventional sdAbs (Ikeuchi et al., 2021), suggesting that we could improve the solubility of sdAbs without substantially compromising thermal stability and folding. In contrast, the W99A mutation in sdAb B CDR3 resulted in an increase of *T*_m_ and ΔH values (Table 3). Considering a previous study indicating that CDRs contribute greatly to the thermal stability of sdAbs via CDR–FR2 interaction (Kinoshita et al., 2022), our DSC results suggest that the W99A mutation in sdAb B CDR3 may affect the CDR–FR2 interaction and thereby improve thermal stability.

**TABLE 3:** Melting temperature (*T*_m_) and ΔH values of sdAb mutants.

	*T*_m_ (°C)	ΔH (kcal/mol)
sdAb A V37Y‐W47L	72.9 ± 0.2	122.3 ± 4.0
sdAb A FR2 YERL	71.3 ± 0.2	129 ± 4.3
sdAb A YERLS	70.1 ± 0.2	129 ± 3.6
sdAb B V37Y‐W47L	58.2 ± 0.0	58.2 ± 3.1
sdAb B FR2 YERL	57.0 ± 0.0	60.0 ± 0.5
sdAb B YERLA	59.7 ± 0.0	69.5 ± 1.0

*Note*: Measurements were conducted in Arg‐containing buffer. Averages and standard deviations of three independent measurements are shown.

**FIGURE 9:**
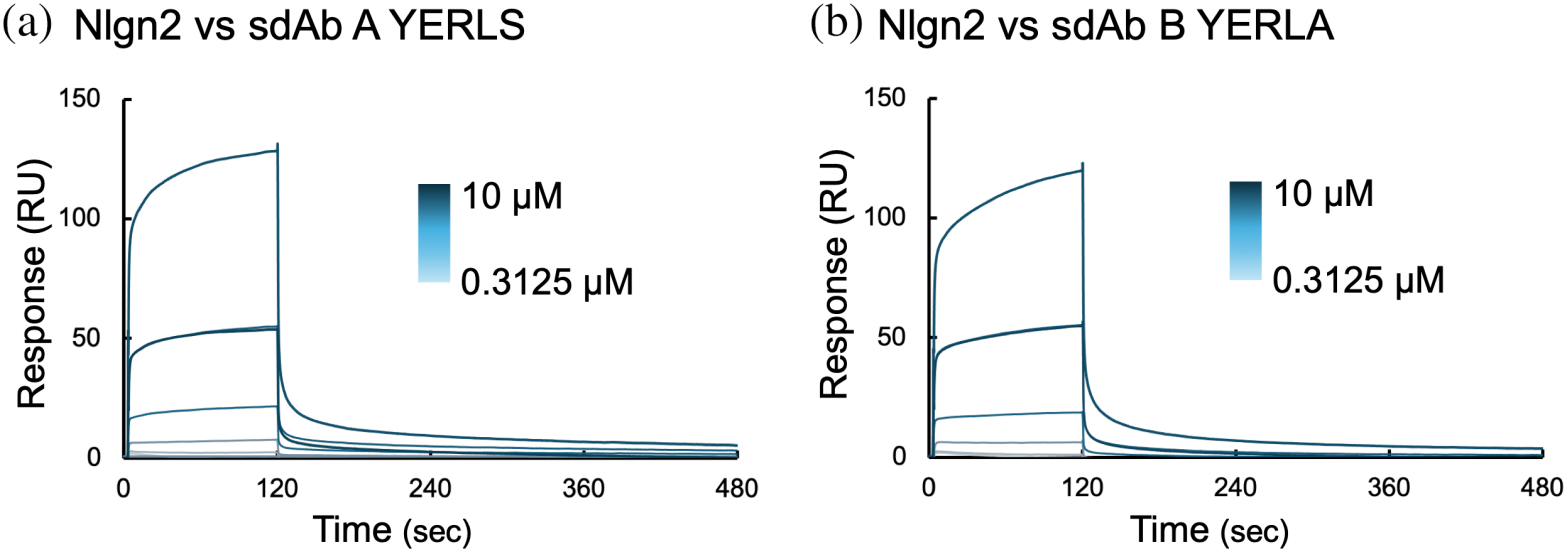
Interaction analyses of sdAbs. The sensor gram obtained by surface plasmon resonance (SPR) for each mutant. (a) sdAb A YERLS. (b) sdAb B YERLA. Representative results from three independent measurements are shown.

Finally, to examine the functionality of the sdAb A and B mutants, we conducted interaction analyses using sdAb A YERLS and sdAb B YERLA and their antigen Nlgn2. We used surface plasmon resonance (SPR) analysis to measure the binding activities of the mutants (Figure 9). The recombinant Nlgn2 extracellular domain was immobilized on a sensor chip, and sdAbs were injected as analytes at concentrations ranging from 0.3125 μM to 10 μM. We observed an increase in the binding response with increasing concentration of the analytes, indicating that the sdAb mutants retained their binding activity toward the antigen. However, we were unable to determine the kinetic parameters due to low affinity. To estimate the effect of mutations on antigen binding, we investigated the affinity of back‐mutated sdAb A V37Y‐W47L by SPR. The sdAb A V37Y‐W47L mutant showed a comparable response in the same concentration range as the sdAb A YERLS mutant (Figure S3), suggesting that the FR2 mutations did not significantly reduce the affinity. Subsequently, to assess the effect of the W99A mutation on sdAb B binding, we attempted to perform SPR analysis using the sdAb B FR2 YERL mutant, which retains the W99 residue in the presence of Arg to maintain the monomeric state, and compared it with sdAb B YERLA. However, the binding response was significantly lower than that for sdAb B YERLA in the absence of Arg, presumably because Arg interfered with the interaction (Figure S4). Nevertheless, both sdAb B YERL and YERLA mutants showed a similar binding profile in the presence of Arg, indicating the intrinsically low affinity of sdAb B even with W99.

## 3 DISCUSSION

In this study, we developed a strategy to enhance the solubility of sdAbs that relies on a sequence‐based approach and a computational approach (i.e., SAP calculation). We remodeled sdAbs possessing typical VH‐like hallmark residues isolated from the alpaca immune library (Figure 1a). It is noteworthy that the *T*_m_ values of engineered sdAb A YERLS and sdAb B YERLA were higher than those of previously reported soluble sdAbs (Vranken et al., 2002) with a VH‐like framework. This suggests that our strategy enhanced the solubility of sdAbs that possess intrinsic high thermal stability together with low solubility.

**FIGURE 10:**
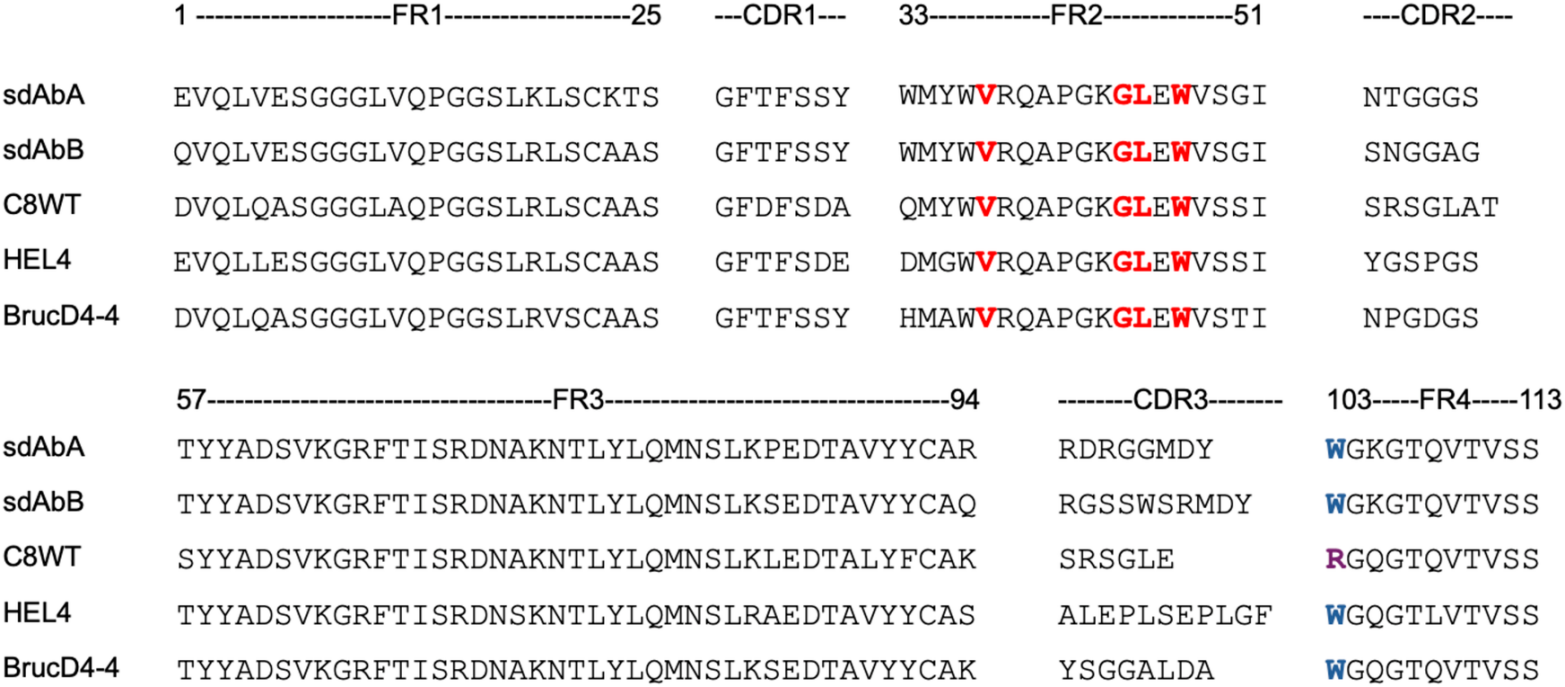
Sequence comparison of sdAbs with VH‐like hallmark residues.

Normally, exposure of VH‐like hallmark residues into the solvent leads to aggregation (Davies & Riechmann, 1994; Ward et al., 1989). Indeed, sdAbs in this study could not be eluted in SEC even in the presence of Arg. On the other hand, previous studies reported that even with VH‐like hallmark residues, several clones were soluble in a monomeric state, suggesting the presence of compatibility between CDR and the VH‐like framework (Jespers et al., 2004; Soler et al., 2021; Vranken et al., 2002). To gain insight into this compatibility, we aligned sequences of our sdAbs with those of reported soluble sdAbs: C8WT (Soler et al., 2021), HEL4 (Jespers et al., 2004), and BrucD4‐4 (Vranken et al., 2002) (Figure 10). These previous studies suggested that various factors contributed to the observed solubility. Soler et al. (2021) suggested that the lack of the highly conserved W103 and the absence of R45, a hallmark residue in the VHH‐like framework, allowed the CDR3 loop to cover the hydrophobic FR2 and thereby suppress aggregation in the case of C8WT (Soler et al., 2021). On the other hand, W47 of HEL4 was located in the hydrophobic cavity formed by residues G35, V37, and A93, which reduced the hydrophobicity of FR2 in spite of the VH‐like sequecnes (Jespers et al., 2004). Compared to those sdAbs, our sdAbs A and B possess W103 and lack G35, which would contribute to their insolubility.

When we attempted to purify WT sdAb A and sdAb B by IMAC, both were eluted in the soluble fraction. Even after filtering using a 0.8 μm pore‐sized filter, the sdAbs would not form aggregates. Aggregates that were trapped by the 0.2 μm pore‐sized filter likely formed during dialysis against the SEC buffer. Although we succeeded in suppressing this aggregation by adding Arg to the SEC buffer (Figure 1c), the sdAbs were not eluted from the SEC column (Figure 1d). Considering that the sdAbs passed through the 0.2 μm pore‐sized filter in the presence of Arg, this indicates that they were absorbed on the column resin. Additionally, the sdAb B FR2 YERL mutant, which contains hydrophobic regions in FR2 YERL and CDR3 according to the SAP calculation, was eluted at more than one column volume position (Figure 2e). This result supports the scenario that exposing the hydrophobic surface to the solution contributes to its interaction with the SEC resin, which is primarily composed of dextran and agarose. Because the exposed hydrophobic surface often non‐specifically interacts with materials, remodeling of the hydrophobic surface is important for the design of functional proteins. Therefore, our SAP‐based resurfacing is a promising strategy for improving the solubility of functional sdAbs, especially for cases in which the hydrophobic surface is derived from residues other than the hallmark residues.

SPR analysis confirmed that the mutants sdAb A YERLS and sdAb B YERLA maintained their binding activity, although the affinity for Nlgn2 was low for both engineered sdAbs (Figure 9). Generally, mutation to the CDR of Abs is not preferred for fear of losing important residues for binding interactions. To create sdAb B YERLA, we mutated W99 in the center of CDR3, which might be involved in the direct interaction with the antigen. Indeed, the binding response for the YERLA mutant was lower than that of the FR2 YERL mutant, suggesting a potential reduction in affinity due to the W99A substitution. Nevertheless, the similarity in the shape of the response curves suggests that the binding affinity would be originally low even with W99. Collectively, the observed low affinity of sdAb B YERLA would not be the result of this mutation.

In conclusion, we suggest a strategy to enhance the solubility of sdAbs that are not stable in solution and that are difficult to analyze. We characterized engineered sdAbs and confirmed that they maintain their functionality. The computational approach is a powerful tool that can improve the physical properties of proteins even in the absence of structural information. We believe that our strategy can be applied not only to sdAbs but also to other proteins.

## 4 METHODS

### 4.1 Expression and purification of recombinant Nlgn2 for immunization

The gene of the extracellular domain of mouse Nlgn2 (15–678) was inserted in pcDNA™3.4 TOPO^R^ vector (Thermo Fisher Scientific, Waltham, MA, USA) with His6 tag in the C‐terminus. Expi293F™ cells (Thermo Fisher Scientific) were used for protein expression. The cells were cultured at 37°C, 125 rpm, and 8% CO_2_. The supernatant was collected 5 days after transfection and filtered through a 0.8 μm pore‐sized filter. After dialysis against the buffer for IMAC (20 mM Tris‐HCl pH 8.0, 500 mM NaCl, and 5 mM Imidazole), the sample was applied to a column filled with Ni‐NTA agarose (QIAGEN, Hilden, Germany) equilibrated with the IMAC buffer. The Nlgn2 extracellular domain with His6 tag in the C‐terminus was eluted with 20 mM Tris‐HCl pH 8.0, 500 mM NaCl, and 200 mM imidazole. The sample was then dialyzed against phosphate buffered saline (PBS) at 4°C overnight followed by SEC using a Hiload 16/600 Superdex 200 pg. column (Cytiva, Marlborough, MA, USA) equilibrated with PBS. The main peak appeared as a dimeric state of Nlgn2 and was collected and concentrated using an Amicon‐Ultra‐15 50 K system (Merck KGaA, Darmstadt, Germany).

### 4.2 Selection of sdAbs

An alpaca was immunized with the recombinant Nlgn2 extracellular domain. Library construction from the peripheral blood B cells obtained from the immunized alpaca and antibody selection was conducted as described in previous studies (Ishii et al., 2021; Yokoo et al., 2022). Briefly, total RNA was obtained using Trizol followed by cDNA synthesis. Antibody genes were amplified by PCR and cloned into a phagemid vector (Barbas III et al., 2001). The library DNA was electroporated into *E. coli* XL‐1 Blue followed by VCS M13 helper phage infection, and phage production was induced in the presence of 1 mM of isopropyl‐1‐thio‐β‐D‐galactopyronoside (IPTG). The phage was precipitated from the bacterial supernatant using PEG/NaCl and resuspended in 1% bovine serum albumin/PBS. The sdAbs were selected by three rounds of biopanning in microtiter wells. Two converged sdAb sequences were identified and cloned, designated as sdAb A and B.

### 4.3 Expression and purification of sdAbs

The genes were cloned in the pRA2 vector (Makabe et al., 2005) with His6 tag in the C‐terminus and a pelB leader sequence at the N‐terminus. Mutants were generated by site‐directed mutagenesis using a KOD One^R^ Mutagenesis Kit (Toyobo, Tokyo, Japan). All sdAbs and mutants were expressed and purified using the same method described in a previous study (Yokoo et al., 2022). The sdAbs were expressed by *E. coli* strain BL21(DE3) transformed with the vector. The cells were grown in 1 L of lysogeny broth (LB) medium supplemented with 100 μg/mL ampicillin at 37°C and 125 rpm. When the optical density at 600 nm reached around 0.8–1.0, protein expression was induced by IPTG at a final concentration of 0.5 mM. After cultivating overnight at 20°C and 95 rpm, the cells were harvested by centrifugation at 7000 × *g* for 10 min, followed by resuspension with 20 mM Tris‐HCl pH 8.0, 500 mM NaCl, and 5 mM imidazole. Resuspended cells were sonicated using an ultrasonic cell disruptor. The soluble fraction was collected as supernatant by centrifugation at 40,000 × *g* for 30 min, filtered through a 0.8 μm pore‐sized filter, and loaded onto Ni‐NTA agarose resin (QIAGEN). The proteins then were eluted with the IMAC elution buffer (20 mM Tris‐HCl pH 8.0, 500 mM NaCl, and 200 mM imidazole) and dialyzed against the SEC buffer (10 mM HEPES‐NaOH pH 7.4, 150 mM NaCl, and 3 mM CaCl_2_) or the Arg buffer (20 mM Tris‐HCl pH 8.0, 200 mM NaCl, and 200 mM Arg‐HCl). The final purification was performed by SEC using a Hiload 16/600 Superdex 75 pg. column (Cytiva). Before loading samples for SEC, they were filtered through a 0.2 μm pore‐sized filter. To check for the presence of soluble proteins, SDS‐PAGE and western blotting were performed. SDS‐PAGE gels were stained with Coomassie brilliant blue (CBB). For western blotting, anti‐His‐tag monoclonal Ab HRP‐DirecT (MBL, Tokyo, Japan), which binds to His6 tag, was used to detect the sdAbs using ECL Western Blotting detection Reagents (Cytiva).

### 4.4 Model structure preparation and MD simulation

The initial structures of WT and FR2 YERL mutants of sdAb A and B were modeled using ColabFold (Mirdita et al., 2022) with AlphaFold2. The predicted structure of each sdAb with the top score was chosen as the initial structure. The initial structures were solvated with TIP 3P water (Jorgensen et al., 1983). MD simulation was performed using GROMACS 2024.1 (van der Spoel et al., 2005) with CHARMM force field (Bjelkmar et al., 2010). The details of the process are described in a previously published study (Yamamoto et al., 2023). Briefly, we defined the rectangular box with 0.15 M NaCl and the initial structure, followed by minimization, NVT equilibration, and NPT equilibration. The simulation involved three independent runs, each starting from the energy minimization step. The simulation was performed for 100 ns. By clustering the centroid structures between 80 and 100 ns, one structure was extracted from each independent run. The Cα‐RMSDs were computed using GROMACS 2024.1 package (Figure S1). In the RMSD calculation, Cα atoms in the CDR and nine Cα atoms in the C‐terminus were excluded.

### 4.5 SAP calculation

The SAP calculation was conducted following a previously described method (Chennamsetty et al., 2009) but using an in‐house script based on CHARMM software (Brooks et al., 2009). Briefly, SAP calculation analyzed spherical zones around each atom in a protein structure, combining SAA measurements with residue‐specific hydrophobicity values normalized to glycine. For each atom, SAP integrated the summed hydrophobicity of neighboring residues within a defined radius (*R*), normalized by the fully exposed SAA of the residue's side chain in a tripeptide reference structure (Chennamsetty et al., 2009; Chennamsetty et al., 2010). We used the centroid structures that were obtained through clustering of MD trajectories for the calculation. Solvent accessible surface area was calculated from the centroid structure and was used for the SAP calculation using the radius (*R* = 10 Å). Subsequently, the SAP value per residue was obtained. We visualized the SAP mapped structures using UCSF Chimera (Pettersen et al., 2004).

### 4.6 Crystallization of sdAbs

For the crystallization of sdAb A YERLS and B YERLA mutants, the protein was purified with buffer containing 10 mM HEPES‐NaOH pH 7.4, 150 mM NaCl, and 3 mM CaCl_2_. The protein was concentrated using Amicon‐Ultra‐15 10 K (Merck KGaA). Crystals of the sdAb A mutant at 8 mg/mL were grown by vapor diffusion using the hanging drop method at 20°C; the sdAb B mutant was crystallized at 15 mg/mL using the same method. The crystallization solution of the sdAb A mutant consisted of 0.2 M ammonium sulfate and 30% PEG 4000, and that of the sdAb B mutant contained 0.1 M HEPES sodium pH 7.5, 2% PEG 400, and 1.5 M ammonium sulfate. The crystals were dipped in the crystal solution, which was supplemented with 15% glycerol for sdAb A YERLS and 30% glycerol for sdAb B YERLA, prior to freezing. Suitable crystals were harvested, flash frozen in liquid nitrogen, and stored in liquid nitrogen until used for data collection.

### 4.7 Data collection, refinement, and analyses

Data were collected in beamlines BL5A and AR‐NW12 at the Photon Factory (Tsukuba, Japan) under cryogenic conditions (100 K). The diffraction images were processed using the XDS (Kabsch, 2010) program and subsequently merged and scaled with the program AIMLESS (Evans & Murshudov, 2013) of the CCP4 suite (Winn et al., 2011). The structures were determined using the PHASER (McCoy et al., 2007) program and the molecular replacement method. The structures predicted by ColabFold (Mirdita et al., 2022) with AlphaFold2 were used for the molecular replacement. The coordinates were refined with the program REFMAC5 (Murshudov et al., 1997) and manually improved with COOT (Emsley et al., 2010). Validation was carried out using PROCHECK (Laskowski et al., 1993). Table 1 provides data collection and structure refinement statistics. Molecular graphics and Cα‐RMSD calculations between structures were performed with UCSF ChimeraX (Meng et al., 2023). In the RMSD calculation, nine Cα atoms in the C‐terminus were excluded. For sdAb A, the average values were calculated from four structures of sdAb A YERLS in the unit.

### 4.8 CD measurements

A JASCO J‐1500 spectropolarimeter (Jasco, Tokyo, Japan) was used to take CD spectroscopy measurements in the far ultraviolet region. The protein sample was placed in a 1‐mm quartz cuvette in 10 mM HEPES‐NaOH pH 7.4, 150 mM NaCl, and 3 mM CaCl_2_ at a concentration of 10 μM. The spectrum shown was the accumulation of five measurements. The spectra were analyzed using the BeStSel webserver (Kardos et al., 2025; Micsonai et al., 2022). Secondary structural components were described as defined by the previous study (Kardos et al., 2025).

### 4.9 DSC analysis

Thermal stability of the sdAb mutants (65.9 μM) was analyzed using a MicroCal PEAQ‐DSC instrument (Malvern Panalytical, Malvern, UK). Samples were scanned at a speed of 1°C/min from 20 to 100°C. For experiments of sdAb A YERLS and sdAb B YERLA (Figure 6b), the samples were measured in 10 mM HEPES‐NaOH pH 7.4, 150 mM NaCl, and 3 mM CaCl_2_. On the other hand, Arg‐containing buffer, 10 mM HEPES‐NaOH pH 7.4, 150 mM NaCl, 3 mM CaCl_2_, and 200 mM Arg‐HCl, was used for measurement to compare thermal stabilities of sdAb A and B V37Y‐W47L, FR2 YERL mutant, sdAb A YERLS, and sdAb B YERLA mutant (Figure 8). *T*_m_ values were calculated with MicroCal PEAQ‐DSC software using a non‐two‐state denaturation model.

### 4.10 SPR analysis

We used a Biacore T200 instrument (Cytiva) for the interaction analysis. For immobilization, we prepared Nlgn2 with Avitag (Fairhead & Howarth, 2015) in the C‐terminus region and biotinylated it with BirA. Nlgn2 with Avitag was eluted as a dimeric peak in SEC, and the buffer (10 mM HEPES‐NaOH pH 7.4, 150 mM NaCl, and 3 mM CaCl_2_) was used for the SEC purification. Biotinylated Nlgn2 was captured on sample flow cells of a Series S Sensor Chip SA (Cytiva) via the interaction between biotin and streptavidin. Biotinylated Nlgn2 was immobilized at 100 nM for 1000 RU. The assay was carried out in 10 mM HEPES‐NaOH pH 7.4, 150 mM NaCl, and 3 mM CaCl_2_ containing 0.005% (v/v) Tween‐20 at 25°C at a flow rate of 30 μL/min (Figure 9, Figure S3). For sdAb B FR2 YERL mutant, measurement was conducted in Arg‐containing buffer, 10 mM HEPES‐NaOH pH 7.4, 150 mM NaCl, 3 mM CaCl_2_, and 50 mM Arg‐HCl containing 0.005% (v/v) Tween‐20. For comparison, sdAb B YERLA mutant was also measured by the same method using the 50 mM Arg‐containing buffer (Figure S4). The binding curves were obtained by subtracting the binding response on the reference flow cells from that on the Nlgn2‐immobilized flow cells. The association time was 120 s, and the dissociation time was 360 s. Regeneration was performed after every cycle with 1 M Arg‐HCl pH 4.4.

#### Accession numbers

The coordinates and structure factors of the sdAb A YERLS mutant (PDB: 9L1K) and sdAb B YERLA mutant (PDB: 9L1J) have been deposited in the Protein Data Bank.

## AUTHOR CONTRIBUTIONS

**Yuta Uto:** Investigation; data curation; formal analysis; writing – original draft; visualization; validation. **Makoto Nakakido:** Conceptualization; supervision; funding acquisition; writing – original draft; project administration. **Takanori Yokoo:** Investigation; data curation; writing – review and editing. **Jorge Fernandez‐Perez:** Investigation; writing – review and editing. **Kevin Entzminger:** Investigation; writing – review and editing; methodology. **Toshiaki Maruyama:** Methodology; investigation; writing – review and editing. **C. J. Okumura:** Methodology; writing – review and editing; resources. **Daisuke Kuroda:** Writing – review and editing; methodology; software; resources. **Jose M. M. Caaveiro:** Investigation; methodology; resources; software; writing – review and editing. **Kouhei Tsumoto:** Funding acquisition; project administration; writing – review and editing; supervision.

## FUNDING INFORMATION

This work was funded in part by the Japan Society for the Promotion of Science (grant no. 21H05090 to M.N. and 20H02531 to K.T.); a Grant‐in‐aid for the Japan Society for the Promotion of Science Fellows (grant no. 23KJ0516 to T.Y.); the Japan Agency for Medical Research and Development (grant no. JP19am0401010h and JP23tk0124002h to M.N.; JP223fa627001 (UTOPIA) and JP223fa727002 (SCARDA) to K.T.); and the Research Support Project for Life Science and Drug Discovery (Basis for Supporting Innovative Drug Discovery and Life Science Research) from the Japan Agency for Medical Research and Development (grant no. JP22ama121033 to K.T.).

## Supporting information


**Figure S1.** shows root‐mean‐squared deviation (RMSD) values of Cα atoms of the framework region (FR) of sdAb A and B FR2 YERL mutants during molecular dynamics (MD) simulation.
**Figure S2.** shows SAP mapped structures of the sdAb A and B WT.
**Figure S3.** shows interaction analysis of sdAb A back‐mutated mutant. The sensor gram obtained by surface plasmon resonance (SPR) for sdAb A V37Y‐W47L (YGLL) mutant.
**Figure S4.** shows interaction analyses of sdAb B mutants in the presence of Arg. The sensor gram obtained by surface plasmon resonance (SPR) for sdAb B YERL and YERLA mutant.

## Data Availability

The data that support the findings of this study are available from the corresponding author upon reasonable request.
