# Supplementary material for: Improving the solubility of single domain antibodies using VH‐like hallmark residues
Source: Protein Sci. 2025 Jun 16;34(7):e70189. doi: 10.1002/pro.70189 (PMC12168134; doi:10.1002/pro.70189)
Supplement: Supplementary file 1 — Figure S1. shows root‐mean‐squared deviation (RMSD) values of Cα atoms of the framework region (FR) of sdAb A and B FR2 YERL mutants during molecular dynamics (MD) simulation. Figure S2. shows SAP mapped structures of the sdAb A and B WT. Figure S3. shows interaction analysis of sdAb A back‐mutated mutant. The sensor gram obtained by surface plasmon resonance (SPR) for sdAb A V37Y‐W47L (YGLL) mutant. Figure S4. shows interaction analyses of sdAb B mutants in the presence of Arg. The sensor gram obtained by surface plasmon resonance (SPR) for sdAb B YERL and YERLA mutant. [file PRO-34-e70189-s001.docx]

***Supporting information***


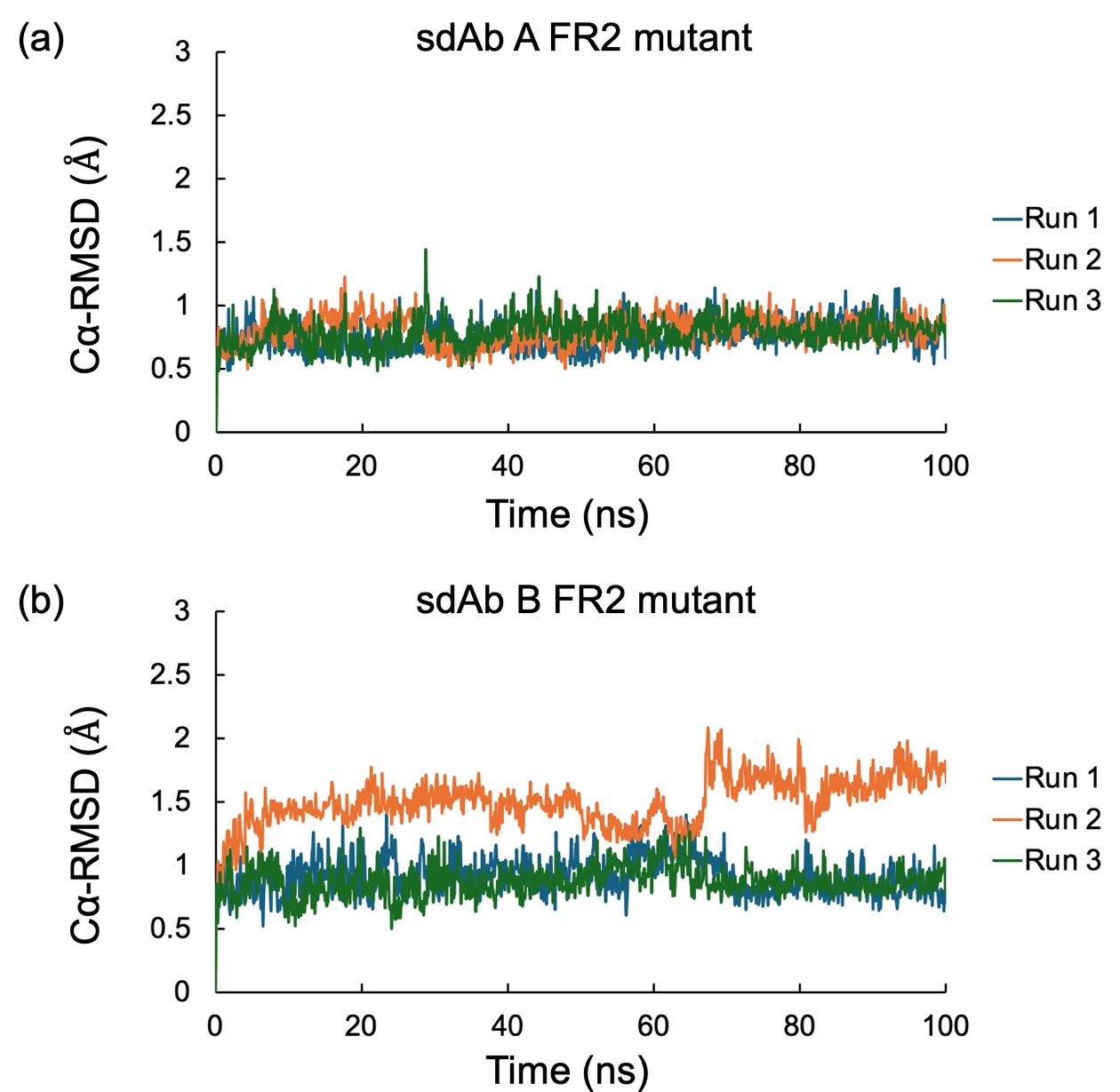


**Figure S1. RMSD values of Cα atoms in the FR of sdAb A and B FR2 mutants.** MD simulations were performed using the predicted structures by AlphaFold2.


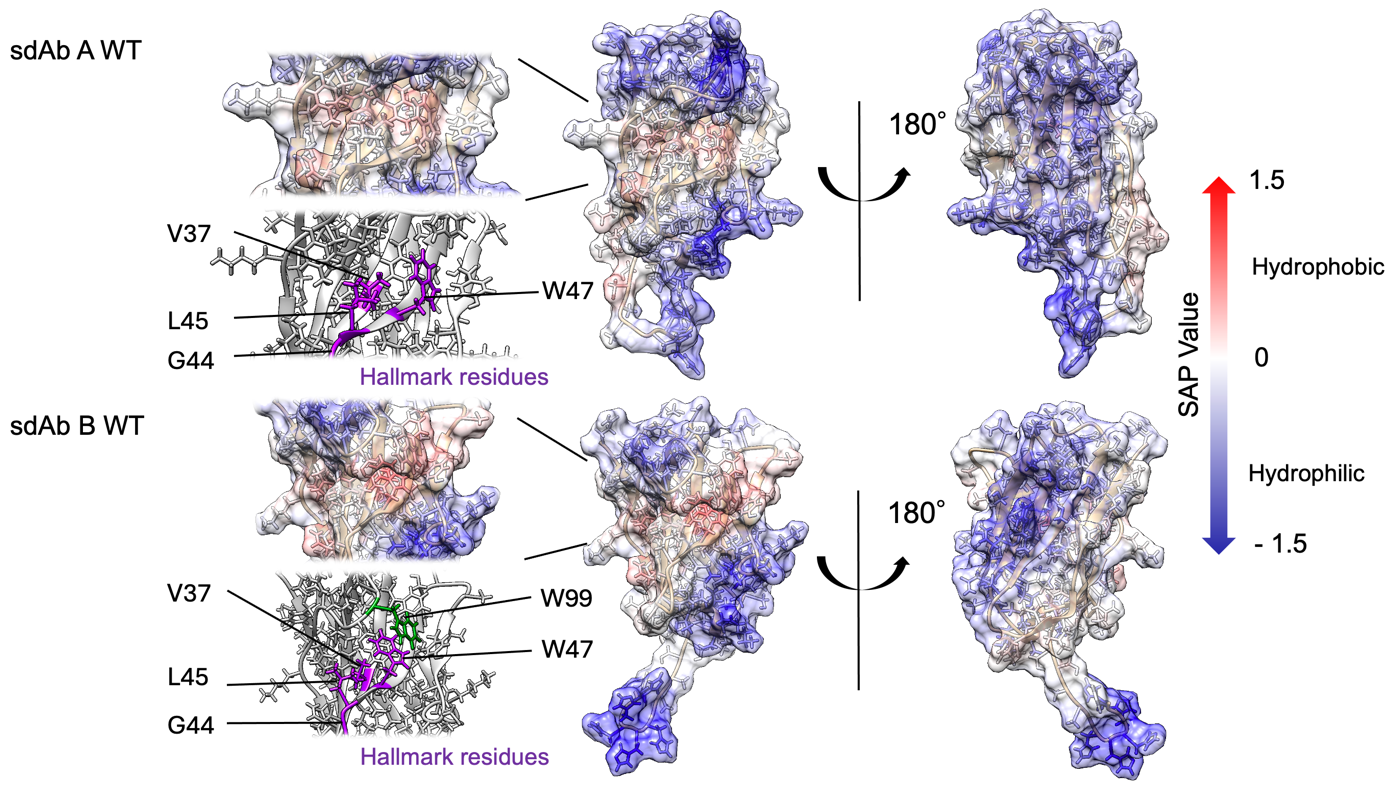


**Figure S2. SAP mapped structures of the sdAb WT.** W99 of sdAb B in the middle of CDR3 is highlighted in green. The hallmark residues are highlighted in purple. The structures are in the similar orientation as Figure 3.

**
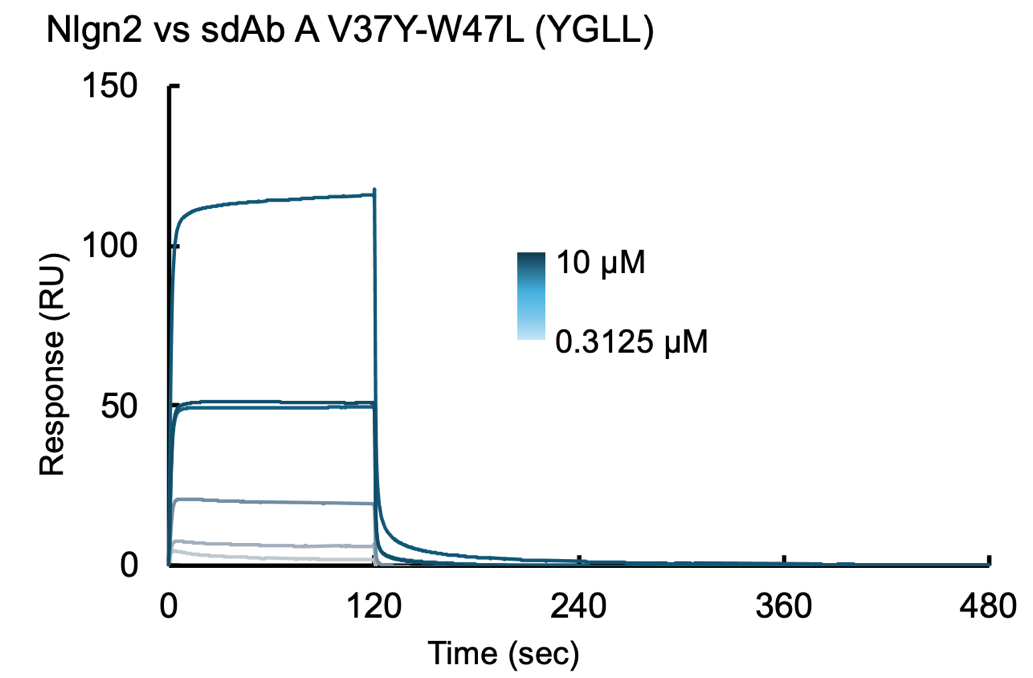
**

**Figure S3. Interaction analysis of sdAb A back-mutated mutant.** The sensor gram obtained by surface plasmon resonance (SPR) for sdAb A V37Y-W47L (YGLL) mutant. Representative result from three independent measurements is shown.


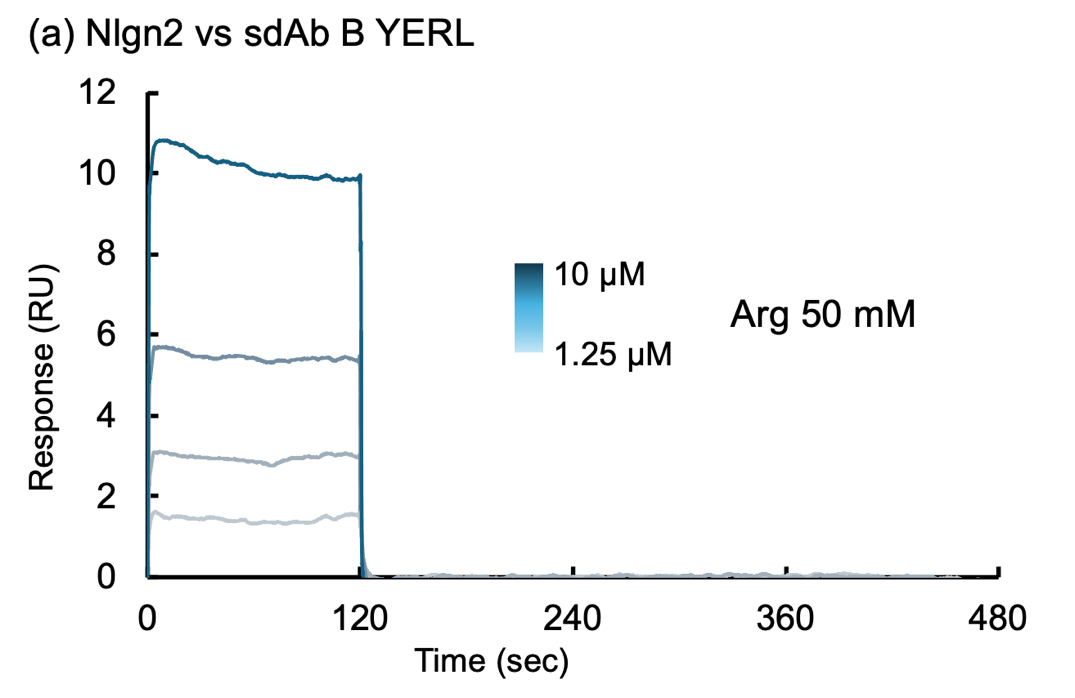


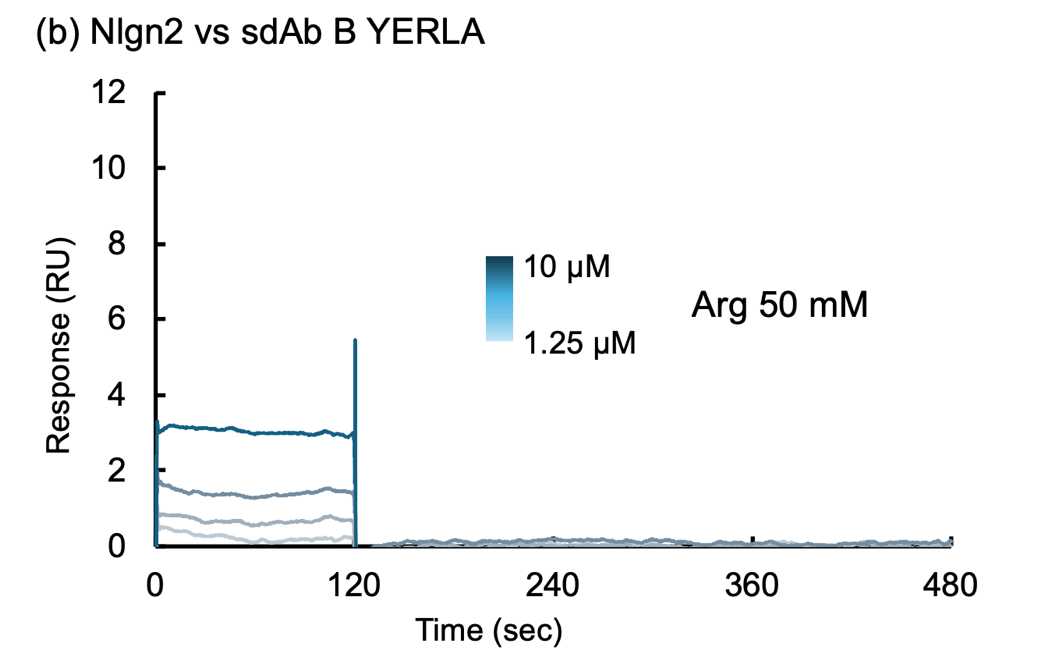


**Figure S4. Interaction analyses of sdAb B mutants.** The sensor gram obtained by surface plasmon resonance (SPR) for sdAb B YERL and YERLA mutant. The experiment was carried out using Arg buffer, containing 50 mM Arg to analyze in monomeric state. Representative results from three independent measurements are shown.
